# Posterior fossa horns; a new calvarial finding of mucopolysaccharidoses with well-known cranial MRI features

**DOI:** 10.3906/sag-1908-70

**Published:** 2020-06-23

**Authors:** Çağrı DAMAR, Betül Emine DERİNKUYU, Muazzez Asburçe Bike Olgaç KILIÇKAYA, Mehmet ÖZTÜRK, Çiğdem ÖZTUNALI, Ayşe Gül ALIMLI, Öznur Leman BOYUNAĞA, Murat UÇAR, Fatih Süheyl EZGÜ, Leyla TÜMER, Alp Özgün BÖRCEK, Ahmet SIĞIRCI

**Affiliations:** 1 Department of Radiology, Faculty of Medicine, Gaziantep University, Gaziantep Turkey; 2 Department of Pediatric Radiology, Dr. Sami Ulus Maternity and Children’s Research and Education Hospital, Ankara Turkey; 3 Department of Pediatrics, Division of Inborn Errors of Metabolism and Nutrition,Dr. Sami Ulus Maternity and Children’s Research and Education Hospital, Ankara Turkey; 4 Department of Radiology, Faculty of Medicine, Selçuk University, Konya Turkey; 5 Department of Radiology, Faculty of Medicine, Osmangazi University, Eskişehir Turkey; 6 Department of Pediatric Radiology, Ministry of Health, Ankara City Hospital, Ankara Turkey; 7 Department of Radiology, Faculty of Medicine, Gazi University, Ankara Turkey; 8 Department of Pediatrics, Division of Inborn Errors of Metabolism and Nutrition, Faculty of Medicine, Gazi University, Ankara Turkey; 9 Department of Neurosurgery, Faculty of Medicine, Gazi University, Ankara Turkey; 10 Department of Radiology, Faculty of Medicine, İnönü University, Malatya Turkey

**Keywords:** Lysosomal storage diseases, glycosaminoglycans, mucopolysaccharidosis, magnetic resonance imaging, internal hypertrophy of the occipitomastoid sutures

## Abstract

**Background/aim:**

Mucopolysaccharidoses (MPS) are a group of hereditary metabolic diseases. The aim of this study was to share the previously unreported calvarial finding of internal hypertrophy of the occipitomastoid sutures (IHOMS) together with some other well-known cranial MRI findings in this patient series.

**Materials and methods:**

A retrospective evaluation was conducted of 80 cranial MRIs of patients who had been diagnosed and followed up with MPS from 2008 to 2019 in our center. Of these patients, 11 had Hurler, 14 had Hunter, 24 had Sanfilippo, 15 had Morquio, 14 had Maroteaux–Lamy, and 2 had Sly disease. The cranial MRIs were assessed in two main groups as parenchymal intradural cranial MRI findings and extradural calvarial findings.

**Results:**

The most common parenchymal intradural cranial MRI findings were white matter signal alterations (n = 51, 63%) and perivascular space enlargements (n = 39, 48%). The most common extradural calvarial findings were J-shaped sella (n = 45, 56%) and tympanic effusion (n = 44, 55%). Although IHOMS was defined in a relatively small number of the patients (n = 12, 15%), the prevalence rate was high in MPS type I (n = 6, 54%).

**Conclusion:**

The abnormal cranial MRI findings of the MPS patients, including the newly identified IHOMS, may provide diagnostic clues to differentiate the type of the disease in radiological imaging.

## 1. Introduction

Lysosomal storage diseases (LSDs) are a group of congenital metabolism errors. They are caused by the accumulation of macromolecules (proteins, polysaccharides, and lipids) due to defects of the enzymes needed for the breakdown of these macromolecules. The substrate accumulation in macrophages or connective tissue cells affects various systems of the body. Mucopolysaccharidoses (MPS) constitute one of the most prevalent subgroups of LSDs, in which the enzymatic defects cause glycosaminoglycans (GAGs) (also called mucopolysaccharides) to accumulate in the skeletal system, cornea, heart, vasculature, and several other organ systems. The GAG fragments increase in the fluids of the body (urine, blood, and cerebral spinal fluid) [1–3].

There are 7 different well-defined types of MPS and some of these also have subtypes (MPS type I: Hurler disease, MPS type II: Hunter disease, MPS type III: Sanfilippo disease, MPS type IV: Morquio disease, MPS type VI: Maroteaux–Lamy disease, MPS type VII: Sly disease, MPS type IX). Except for MPS type II disease, which has an X-linked recessive inheritance, all other MPS types are transmitted in an autosomal recessive fashion. Patients with MPS generally look normal at birth and they may lack the characteristic features of the disease at that time. However, they are often affected by an abnormal, multisystemic, progressive developmental process that varies according to the type and the severity of the disease. As a result, a variety of skeletal manifestations called dysostosis multiplex (DMX) and extraskeletal manifestations with some phenotypic abnormalities peculiar to MPS occur clinically [1–3].

Although the involved enzymes and the substances accumulated due to their deficiencies have been clearly specified for each type of MPS, the mechanisms resulting in disease symptoms, especially for skeletal manifestations, have not been clearly identified. Skeletal symptoms usually appear first in most MPS, except for MPS type III, in which skeletal symptoms become apparent later after other system manifestations, mainly in the central nervous system (CNS). They generally occur due to the effects of the primary catabolic defect in the bones and cartilage. The skeletal manifestations of MPS depend on the type of the disease and include short stature, atlantoaxial instability (especially in MPS type IV), spinal deformations (gibbus deformity, scoliosis, kyphosis, and platyspondyly), spinal cord compression, joint stiffness and contractures, joint laxity (specific to MPS type IV), macrocephaly, hip dysplasia, valgus deformity of the knees, chest deformity, and joint effusions. MPS type III is characterized by fewer skeletal abnormalities that are accompanied by severe neurological manifestations. The radiological features of DMX include J-shaped sella turcica, calvarial thickening, shortening of the sternum, broad and short clavicles, platyspondyly, inferiorly beaked vertebrae, dysplastic odontoid process, round iliac wings, hip dysplasia, proximal pointing of short and hypoplastic metacarpals, and hypoplastic thick and short epiphyses of the long bones. Other expected clinical manifestations of MPS include coarse facial features, short stature, arthropathy, visceromegaly, cataracts, and psychomotor retardation [1,4–8].

In the present study, an overview is presented of the most frequent cranial MRI findings of MPS types. Enzyme deficiencies, accumulated metabolites, the presence of DMX, and the main clinical findings are summarized in Table 1 [1,3,7–11].

**Table 1:** Summary of the important features of the MPS types.

Disease type		Deficient enzyme	Accumulatedmetabolites	Radiographic and clinical findings							
				DMX	SS	CFF	OM	JS	CRD	CC	MR
Type I (Hurler)	H	Alpha-L-iduronidase	hs, ds	+	+	+	+	+	+	+	+
	HS/S			Patient exhibits these findings moderate(HS)+/mild(S)+							+/- !
Type II (Hunter) severe/mild		Iduronate sulfatase	hs, ds	+	+	+	+	+	+	-	+/-
Type III (Sanfilippo)	A	Heparan sulfamidase	hs	Patient exhibits these findings -/mild +						-	+~
	B	N-acetyl-glucosaminidase									
	C	Acetyl-CoA: alpha-glucosaminideAcetyltransferase									
	D	N-acetylglucosamine6-sulfatase									
Type IV (Morquio)	A	Galactose-6-sulfatesulfatase	ks, cs	+	+ #	-/+	-/+	+ *	+	+	- !
	B	Beta-galactosidase									
Type VI (Maroteaux–Lamy)		N-acetylgalactosamine-4-sulfatase	ds	+	+	+	+	+	+	+	- !
Type VII (Sly)		Beta-glucuronidase	hs, ds, cs	+	+	-/+	+	+	+	+	-/+

H: Hurler, HS: Hurler–Schie, S: Schie, hs: Heparan sulfate, ds: Dermatan sulfate, ks: Keratan sulfate, cs: Chondroitin sulfate, DMX: Dysostosis multiplex, SS: Short stature (#: evident due to platyspondyly), CFF: Coarse facial features, OM: Organomegaly (hepatomegaly and/or splenomegaly), JS: Joint stiffness (*: stiffness and laxity), CRD: Cardiorespiratory disorders, CC: Corneal clouding, MR: Mental retardation [!: patients exhibit normal intelligence with MPS type 1 S (Schei), type IV, and type VI], ~: Mental retardation and/or hyperactivity

The aim of our study was to share some prominent cranial MRI findings of different MPS types and to show an unreported calvarial finding of posterior fossa in addition to some other well-known MRI findings in patients with MPS. 

## 2. Patients and methods

The definitive diagnosis of MPS was based on a positive family history and the presence of specific phenotypic and clinical features, determination of elevated GAG levels in urine samples and skeletal surveys positive for DMX, and enzymatic analyses for the detection of specific enzyme deficiencies and identification of the type of the disease. All patients who had been diagnosed with MPS between 2008 and 2019 at our institution’s Division of Pediatric Metabolism were retrospectively identified from the electronic database of patient records. 

Of these, 80 patients (male and female) who had pretreatment baseline MRIs of the cranium were included in the present study. Most of the cranial MRI scans were performed in our institution. Some of the patient MRIs recorded in our picture archiving and communication system (PACS) were performed in different centers or institutions.

Eleven patients in this study had Hurler disease (type I), 14 had Hunter disease (type II), 24 had Sanfilippo disease (type III), 15 had Morquio disease (type IV), 14 had Maroteaux–Lamy disease (type VI), and 2 had Sly (VII) disease. At the time of the MRI examinations, patient ages ranged between 1 month and 19 years, with a mean age of 8 years. Some of the patients with type I, II, and VI diseases were in good mental condition, and received enzyme replacement therapies periodically. 

Through the PACS, the cranial MRIs of a total of 80 patients were reviewed retrospectively. The radiological assessment and measurements were performed with the consensus of two radiologists, each with more than 5 years of neuroradiology experience who were blinded to the type of disease. 

Although the images were taken on different 1.5 Tesla MRI machines, all the cranial MRI examinations included basal sequences. Cranial MRI examinations included axial 3-mm-thick spin echo T1-weighted (232 × 320 matrix, TE/TR: 11/447), T2-weighted 261 × 320 matrix, TE/TR: 113/4540), FLAIR (217 × 320 matrix, TE/TI/TR: 92/2500/9000), sagittal 4-mm-thick T1-weighted (248 × 320 matrix, TE/TR: 10/390), and coronal 4-mm-thick T2-weighted (254 × 384 matrix, TE/TR: 95/5690) sequences. 

The assessment of the cranial MRI findings was performed by categorizing the findings into 2 groups. One group of findings included the intradural and parenchymal abnormalities, while the other group included the extradural and calvarial changes. The specific imaging findings evaluated under the heading of each group were as follows:

Group I. MRI findings of intradural and parenchymal changes: parenchymal atrophy, hydrocephalus, white matter signal alterations (WMSAs), perivascular space (Virchow–Robin space) enlargements (PVSEs) in the peri-supraventricular white matter and corpus callosum, optic nerve sheath enlargement (uni-/bilaterally). 

For statistical evaluation, the patients who had type I, II, and VI diseases were divided into three age groups. The first group comprised patients aged ≤2 years, the second group patients were aged 2–10 years, and the third group patients were older than 10 years. The number of the PVSEs in the peri-supraventricular white matter and corpus callosum were also categorized in 3 groups as ≤5, 5–10, and >10.

The Evans ratio was used to define the presence of hydrocephalus and a value of ≥0.30 was accepted as positive for the presence of ventriculomegaly.

Parenchymal atrophy was accepted as present when extensive widening of the Sylvian fissures, brain sulci, and enlargement of other peripheral subarachnoid spaces were observed. Due to the presence of accompanying hydrocephalus in some cases and the presence of morphological asymmetry of sulci and fissures, no measuring systems were used for the scoring of the sulcal widening or subarachnoid space enlargement. Thus, the atrophic changes were subjectively assessed in consensus, as present or absent. 

Group II. MRI findings of extradural and calvarial changes: J-shaped sella (JSS), calvarial thickening, uni-/bilateral tympanic effusion, concave remodeling of the greater wings of the sphenoid bone (RGW), concave remodeling of the cribriform plate of the ethmoid bone (RCP), and internal hypertrophy of the occipitomastoid sutures (IHOMS) uni-/bilaterally. 

The study was approved by the Clinical Trials Ethics Committee. All procedures were in accordance with the 1964 Helsinki Declaration and its later amendments or comparable ethical standards. Informed consent was obtained from all parents of the individual participants in the study.

### 2.1. Statistical analysis

MPS types and related cranial and MR findings were presented as numbers and percentages.

Chi-square analysis was applied to assess the relationship between the age groups of the patients with type I, II, and VI diseases and the number of PVSEs in the perisupraventricular white matter and corpus callosum, and the relationship between IHOMS and other calvarial findings, such as RGW and RCP and calvarial thickening. SPSS 22.0 was used for the statistical analysis (IBM Corp., Armonk, NY, USA) and a value of P < 0.05 was considered statistically significant.

## 3. Results

With the exception of one patient, all cranial MR studies of the patients in the study exhibited some degree of neuroimaging abnormalities. 

Group I: The most frequent cranial intradural and parenchymal MRI findings were WMSAs and PVSEs, which were present in 51 (63%) and 39 (48%) of 80 patients, respectively. There were also findings of parenchymal atrophy (n = 33), hydrocephalus (n = 18), and uni-/bilateral optic nerve sheath enlargement (n = 15) (Table 2). 

**Table 2:** MRI findings of intradural and parenchymal changes.

MPS type (n = 80)	PA	HC	WMSAs	PVSEs	ONSE
Type I (11)	8/11	5/11	10/11	9/11	1/11
Type II (14)	7/14	3/14	11/14	13/14	3/14
Type III (24)	13/24	6/24	16/24	8/24 *	1/24
Type IV (15)	1/15	1/15	2/15	0	3/15
Type VI (14)	2/14	3/14	10/14	9/14	7/14
Type VII (2)	2/2	0	2/2	0	0
Total	33/80	18/80	51/80	39/80	15/80

PA: Parenchymal atrophy, HC: Hydrocephalus, WMSAs: White matter signal alterations, PVSEs: Perivascular space enlargements, *: number of total foci ≤ 5, ONSE: Optic nerve sheath enlargement (unilateral or bilateral).

No relationship was determined between the age groups of the patients with type I, II, and VI diseases and the number of PVSEs (P > 0.05).

Group II: The most frequent bone abnormalities detected on the cranial MR scans were JSS and obliteration of the mastoid air cells and/or the tympanic cavity with mucosal inflammatory changes. These were determined in 45 (56%) and 44 (55%) of the 80 patients, respectively. The other bone and soft tissue abnormalities observed were RCP (n = 23), calvarial thickening (n = 18), uni-/bilateral RGW (n = 11), and unilateral (n = 3) or bilateral (n = 9) IHOMS (Table 3).

**Table 3:** MRI findings of extradural and calvarial changes.

MPS type (n = 80)	JSS	CT	TE	RGW	RCP	IHOMS
Type I (11)	11/11	0	9/11	3/11	6/11	6/11
Type II (14)	11/14	1/14	9/14	3/14	6/14	4/14
Type III (24)	3/24	16/24	7/24	1/24	3/24	0
Type IV (15)	7/15	1/15	9/15	0	0	0
Type VI (14)	12/14	0	9/14	4/14	8/14	2/14
Type VII (2)	1/2	0	1/2	0	0	0
Total	45/80	18/80	44/80	11/80	23/80	12/80

JSS: J-shaped sella, CT: Calvarial thickening, TE: Tympanic effusion (unilateral or bilateral), RGW: (Concave) remodeling of the greater wings (of the sphenoid bone), RCP: (Concave) remodeling of the cribriform plate (of the ethmoid bone), IHOMS: Internal hypertrophy of the occipitomastoid sutures. Note: Three of 12 patients have unilateral and the remaining patients have bilateral IHOMS.

IHOMS was determined in 6 patients with MPS type I, 4 with MPS type II, and 2 with MPS type VI. Although there was no relationship between the IHOMS and other calvarial findings, such as RCP or uni-/bilateral RGW in the patients with type I, II, and VI diseases (P > 0.05), the prevalence rate was high in MPS type I (n = 6, 54%). There was no statistically significant association between IHOMS and calvarial thickening. 

Meckel’s cave invagination, pineal cysts, choroid plexus cysts, posterior fossa arachnoid cysts, mega cisterna magna, and cerebellar vermian hypoplasia were among the other MRI findings encountered.

The cranial MRIs of a newly diagnosed 13-year-old girl with type III MPS revealed no imaging abnormalities. This patient had hyperactivity and mental retardation and exhibited mild somatic features. She had been followed up in the pediatric neurology department due to attention deficit hyperactivity disorder and Rett syndrome for several years before being admitted to the Pediatric Metabolic Diseases Clinic.

## 4. Discussion

In the present study, the cranial MRIs of 80 MPS patients with various disease types were evaluated. The patient data were analyzed in terms of the radiopathological changes described above.

The cranial imaging features of MPS and the effectiveness of various treatment options have been widely described in the literature [12–14]. 

In severe forms of MPS, CNS involvement is always present and the resultant progressive neurodegeneration may lead to the death of these patients in their first or second decades. Patients with mild or moderate forms of the disease live longer but the morbidity rates remain high. Evident mental abnormalities are not expected in mildly expressed forms of MPS I (e.g., in Hurler/Scheie diseases, which show slowly progressive CNS involvement, and in Scheie disease, which almost always exhibits no mental retardation), MPS II (the attenuated phenotype shows no mental deterioration and an intermediate form exhibits slowly progressive CNS involvement), MPS III (a slowly progressive form), MPS IV (Morquio disease), and MPS VI (Maroteaux–Lamy disease) [15].

One of the most frequent abnormalities observed was patchy or diffuse cerebral WMSAs that varied in form and severity in different types of disease (n = 51, 63%). These signal abnormalities have been reported to be due to abnormal myelination and/or gliosis and they become more evident during the course of the disease [16,17]. WMSAs were prominent in type I, II, and most of MPS type III and VI patients in this series. The white matter regions affected were mainly the posterior periventricular white matter areas, and subcortical white matter areas were also affected to a lesser extent. Diffuse periventricular WMSAs in type III disease and patchy WMSAs in other types of MPS have been previously reported [13]. The observations of the current series were compatible with these findings. However, the relationship of patchy or diffuse cerebral WMSAs with clinical findings is a controversial issue in the literature, but a positive association between the extent of white matter lesions and duration of the disease has been reported [18]. 

**Figure 1:**
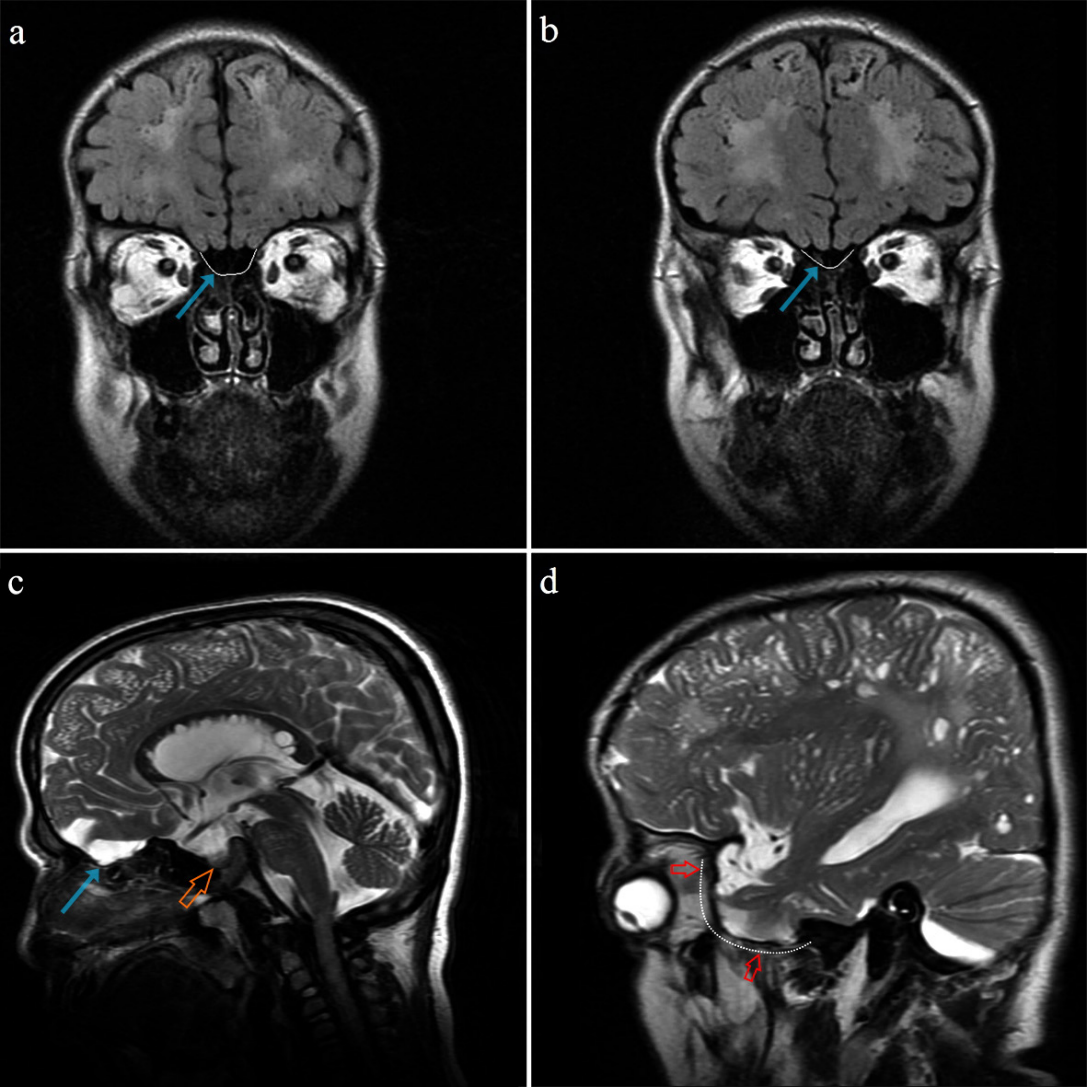
Coronal FLAIR (a, b) and sagittal T2 weighted (c, d) MR images demonstrate concave remodeling of ethmoid roof (blue arrows) and greater wing of the sphenoid bone (red arrows). There is also a ‘J shaped sella’ form (on image c) shown with an orange arrow (15 years old, MPS II).

Perivascular space (also called the Virchow–Robin space) enlargement (PVSE) was one of the most frequently encountered findings in this series (n = 39, 48%). This common but nonspecific feature may be seen in other numerous diseases, as well as in healthy people. The prominent perivascular spaces are classically found to be located in three brain regions: the inferior parts of the basal ganglia, the centrum semiovale, and the midbrain. However, when compared to healthy individuals, some gliotic foci around the enlarged perivascular spaces are present in MPS patients. PVSEs in MPS patients have been related to GAG storage around the perivascular areas or leptomeninges, with a resultant decrease in cerebrospinal fluid (CSF) absorption [14,16,19]. In the current series, PVSEs were usually located in the corpus callosum and/or posterior periventricular white matter, with diameters mostly <10 mm. The patients with MPS type I, II, and VI had a higher incidence of PVSEs. No relationship was determined between the age groups of these patients and the number of PVSEs. Although PVSE was not a prominent feature of type III patients in this series, ≤5 foci were mostly determined in the corpus callosum in 8 of the 24 patients in this group. Furthermore, no PVSEs were observed in type IV patients and in some of the patients with other MPS types (Table 2). In a 15-year-old male patient with type II MPS, PVSEs were more widespread and they were located in the basal ganglia, in the thalamic regions, and in the subcortical areas, resulting in an extensive cribriform appearance of the brain parenchyma. This patient also had patchy WMSAs extending towards the subcortical areas, where the short association fibers (also called U-fibers) are located (Figures 1a–1d). Although PVSEs may not develop in some MPS patients, enlarged PVSs should be considered as evidence for the diagnosis of MPS when accompanied by other specific imaging findings, especially when these are located in the corpus callosum [14]. A previous study comparing patients with MPS and cognitive decline and patients with MPS but without cognitive decline found no significant difference between the groups in the frequency of enlarged perivascular spaces [20]. 

Parenchymal atrophy (n = 33, 41%) and communicating hydrocephalus (n = 18, 22%) were the other two important and significant features in this series. These findings were especially prominent in type I, II, and particularly type III patients (Table 2). In some of the patients with both hydrocephalus and parenchymal atrophy, one of these findings (either hydrocephalus or parenchymal atrophy) was usually more prominent. Currently proposed reasons for the mechanism of hydrocephalus development in MPS patients include the presence of abnormal CSF absorption and venous hypertension [13,14]. The systemic accumulation of GAGs also affects the meninges and impairs the function of arachnoid granulations, thereby decreasing CSF reabsorption, and the abnormal bone proliferation at the skull base decreases cerebral venous outflow. One or both mechanisms lead to communicating hydrocephalus. In the present study, one patient with type VI MPS had noncommunicating hydrocephalus with accompanying periventricular edema that resulted from severe spinal narrowing at C1–2 levels. This patient had undergone surgical treatment and is now progressing well. In respect of hydrocephalus, not only was the supratentorial ventricular system found to be affected in the present study, but the peripheral CSF spaces were also enlarged in some patients. Of these patients, especially those with MPS type I had bitemporal subarachnoid space enlargements and arachnoid cysts in the middle cranial fossa, anterior to the temporal poles.

**Figure 2:**
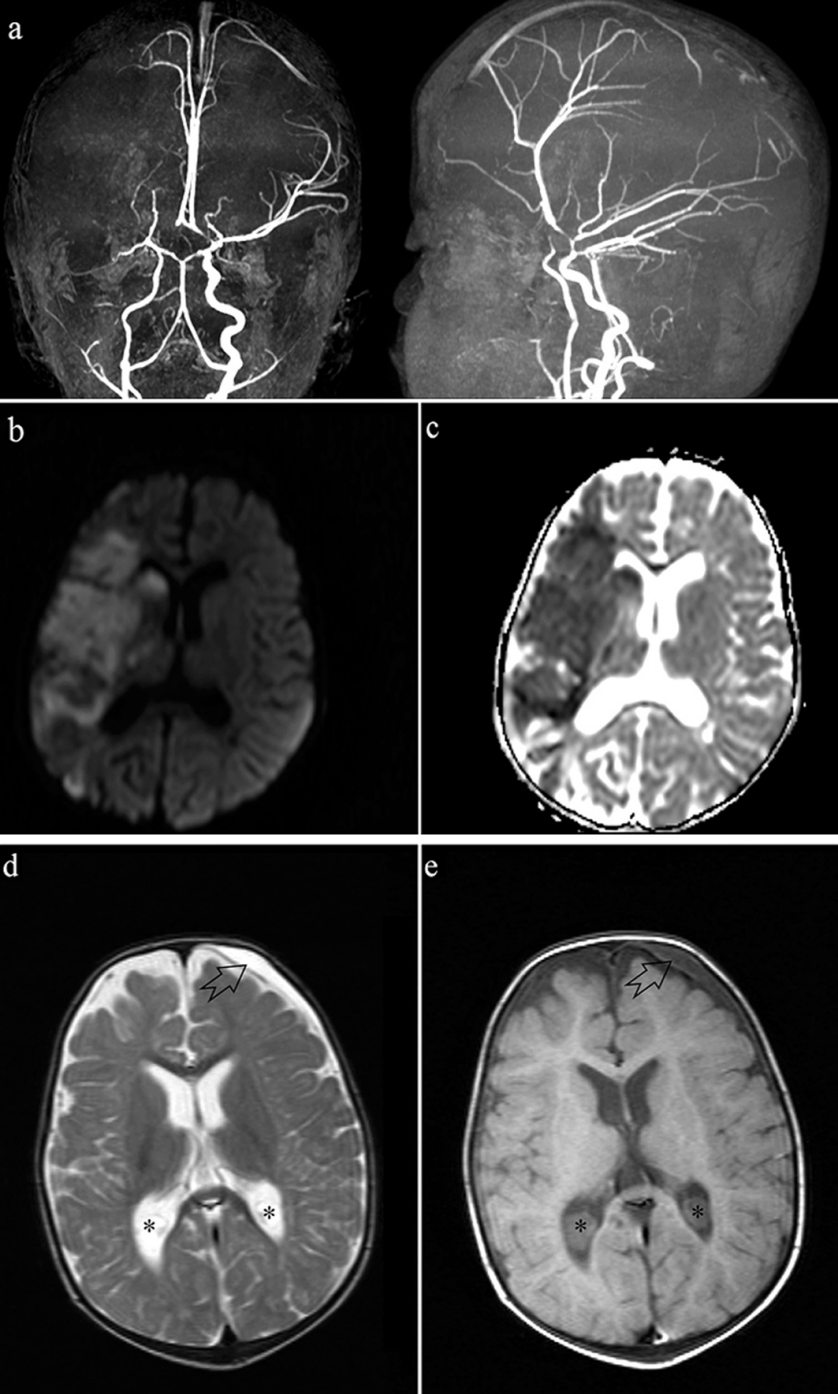
3-D MR angiography images in 2 planes (a) show the absence of right middle cerebral artery (MCA) and right anterior cerebral artery (ACA) flows. Other polygon arteries including the right carotid artery have irregular outlines and focal-segmental stenosis. Diffusion weighted imaging (DWI, B = 1000) (b) and apparent diffusion coefficient (ADC) (c) map images demonstrate a large acute infarction in the right MCA territory (One of 8-month-old twin siblings, MPS type I). Axial T2 (d) and T1 (e) weighted MR images reveal that there is an old subdural hemorrhage anterior to the left frontal lobe (black arrow). There are also bilateral choroid plexus cysts (asterisks) (16 months old, MPS type I).

Of note, 2 patients with type I MPS in this study had the signs of a cerebrovascular accident: an 8-month-old female patient, who was one of identical twins, had right middle cerebral artery infarction with severe encephalomalacia of the right cerebral hemisphere (Figures 2a–2c). The other was a 16-month-old male patient with macrocephaly and bifrontal subarachnoid space enlargements, who also had a left frontal subdural hematoma, which may have resulted from the vasculopathic changes that may be seen in these patients (Figures 2d–2e) [21].

In the cranial MRI evaluations of the present study, the most frequent bone abnormality was JSS (n = 45, 56%). This nonspecific finding may be seen as a normal variant in healthy individuals but it may also be a sign of some disease processes, such as chronic hydrocephalus, optic glioma, osteogenesis imperfecta, achondroplasia, neurofibromatosis, and MPS [22]. JSS has been described in type I, II, IIIA, IV, and VI MPS and it is usually associated with growth hormone deficiency (Figure 1c) [13].

The second most common bone abnormality was uni-/bilateral mastoid inflammatory changes and tympanic effusion (n = 44, 55%). Chronic otitis media may lead to hearing loss in MPS patients [23]. 

**Figure 3:**
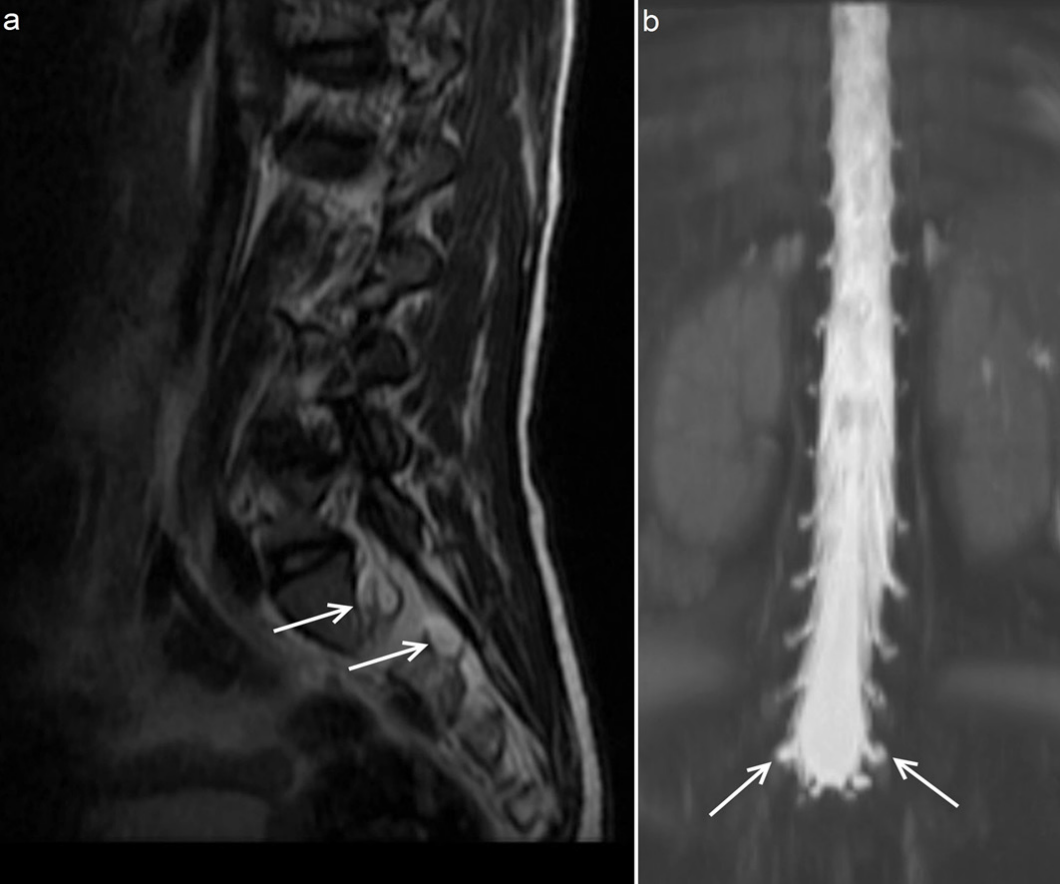
Sagittal T2 weighted MR image (a) and 3-D MR myelography (b) demonstrate multilevel lumbosacral perineural cysts settled in the intervertebral foramina (white arrows) (17 years old, MPS type II).

Other findings include the concave remodeling of the bones of the anterior and middle cranial fossae, resulting in closed meningoencephaloceles [17]. RCP was determined in 23 of the 80 patients in the present study (none with type IV disease). Of the 23 patients with type I, II and VI diseases, 11 also had uni-/bilateral RGW, and all of these patients had meningeal pouches filled with CSF, with or without accompanying cerebral parenchyma extending into the cavity. In addition to the above-mentioned features, there were millimetric leptomeningeal indentations in the calvarium of some patients. Protrusion of the meninges and CSF through the cavum trigeminale (Meckel’s cave), also known as petrous apex cephaloceles, was seen in 9 patients. This condition has been related to the presence of chronic intracranial hypertension [17]. In addition, the MR myelography (maximum intensity projection) and thoracolumbar MR scans of some MPS patients with concave remodeling of the cranial bones showed that bilateral lumbosacral perineural cysts were settled in the intervertebral foramina, proximal to the posterior root ganglia. This additional finding of lumbosacral perineural cysts may support the above-mentioned theory proposing the presence of chronic increase in pressure in different CSF compartments of the CNS in these patients (Figure 3). 

**Figure 4:**
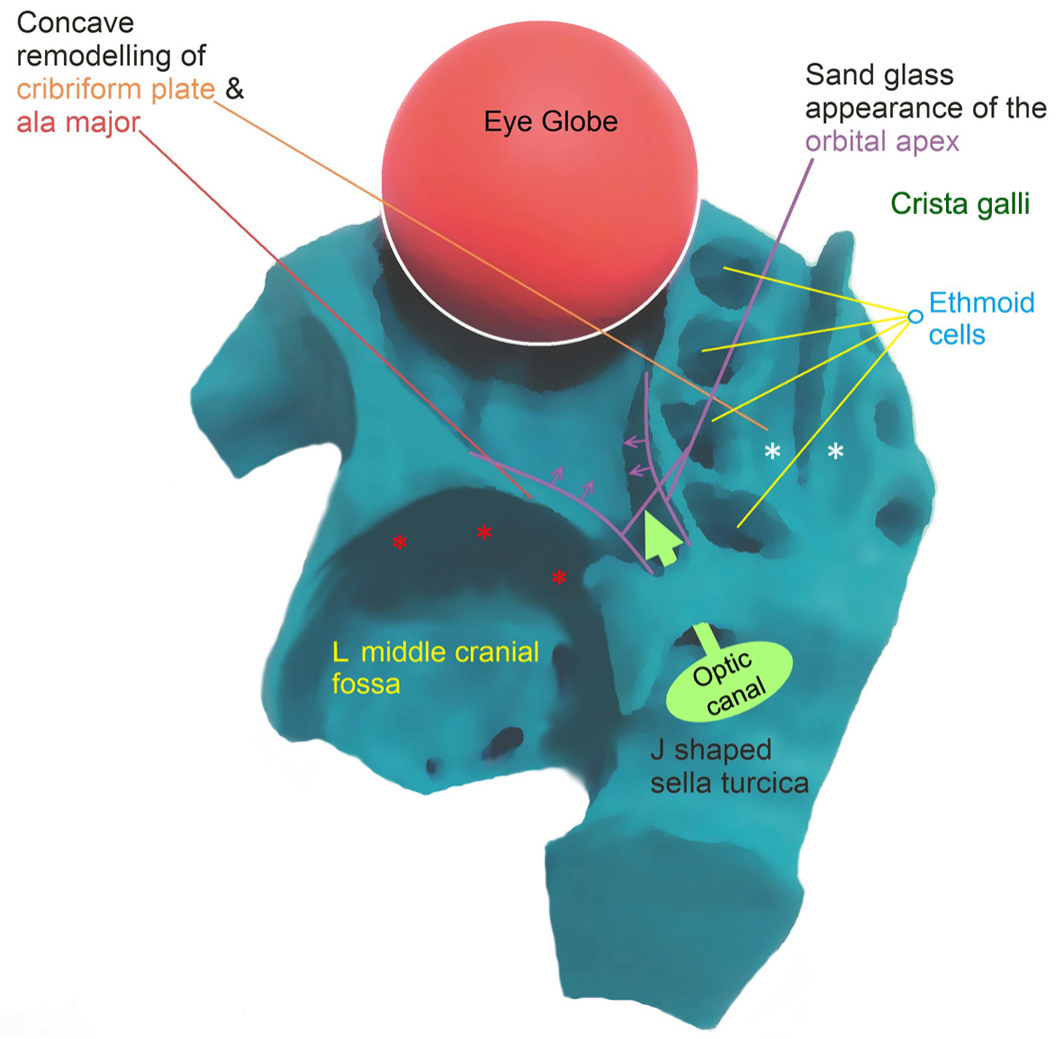
A 3-D illustration orbit of a MPS patient to depict the possible mechanism of pseudoexophthalmos. Sandglass appearance of the orbital apex due to concave remodeling of cribriform plate (white asterisks) and greater wing of the sphenoid bone (red asterisks)

**Figure 5:**
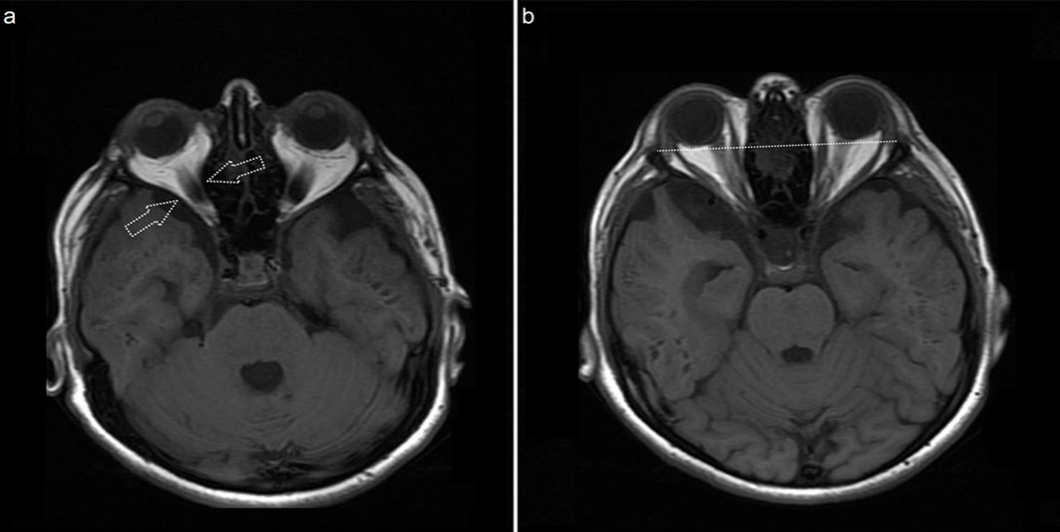
Axial T1 weighted MR image (a) depicts that the orbital apex is narrowed bilaterally and formed as a sandglass appearance (white arrows). Axial T1 weighted MR image (b) reveals bilateral exophthalmos (white dotted line) (15 years old, MPS type II).

Progressive pseudoexophthalmos is another clinical entity that has been reported to be associated with MPS and it is characterized by the presence of shallow orbits [11]. There is currently no generally accepted pathophysiological model to explain this entity. It can be considered that the concave remodeling, both in the greater wings of the sphenoid bone and the cribriform plate of the ethmoid bone, could be the cause of the observed pseudoexophthalmos in these patients. The greater wings of sphenoid bone form the posterolateral parts of the orbit and the presence of bone dysplasia may cause the globe to be displaced anteriorly. In particular, in the inferior orbital plane on axial images, the apical parts of the orbits were observed to be narrowed mediolaterally, with a sandglass appearance. This finding seems to be related to the changes in the greater wings of the sphenoid bone and to the lateral wall convexity of the enlarged ethmoid sinus cells. The RCP also separates the right and the left group of ethmoid sinus cells from each other and displaces them laterally, thereby contributing to the narrowing of the medial apical part of the orbits (Figures 4 and 5). Another finding accompanying pseudoexophthalmos appearance was extraconal fat tissue deposition anteriorly. In a previous ophthalmology study, one of the sonographic morphological changes was widening of the optic nerve and its sheath in type I, II, and VI MPS patients. Increased intracranial hypertension or meningeal GAG storage were suggested to be the cause of the optic nerve sheath enlargement [24,25]. This finding may also relate to communicating hydrocephalus [14]. Widening of the CSF space in the optic nerve sheath may contribute to progressive pseudoexophthalmos. Distinct uni-/bilateral optic nerve sheath enlargement was observed in 15 of the 80 MPS patients (Table 2). In only 3 of these 15 patients was associated hydrocephalus observed. This result may be due to the nonhomogeneous increase in pressure in the CSF compartments. 

In younger patients, who were diagnosed and imaged in the early course of the disease, some of the mentioned features were not prominent. In the MRI studies of 20 patients who were younger than 3 years old, neither significant RGW nor RCP was determined. This finding suggests that some of the changes related to the cranial bone morphology are more likely to develop in the later course of the disease.

**Figure 6:**
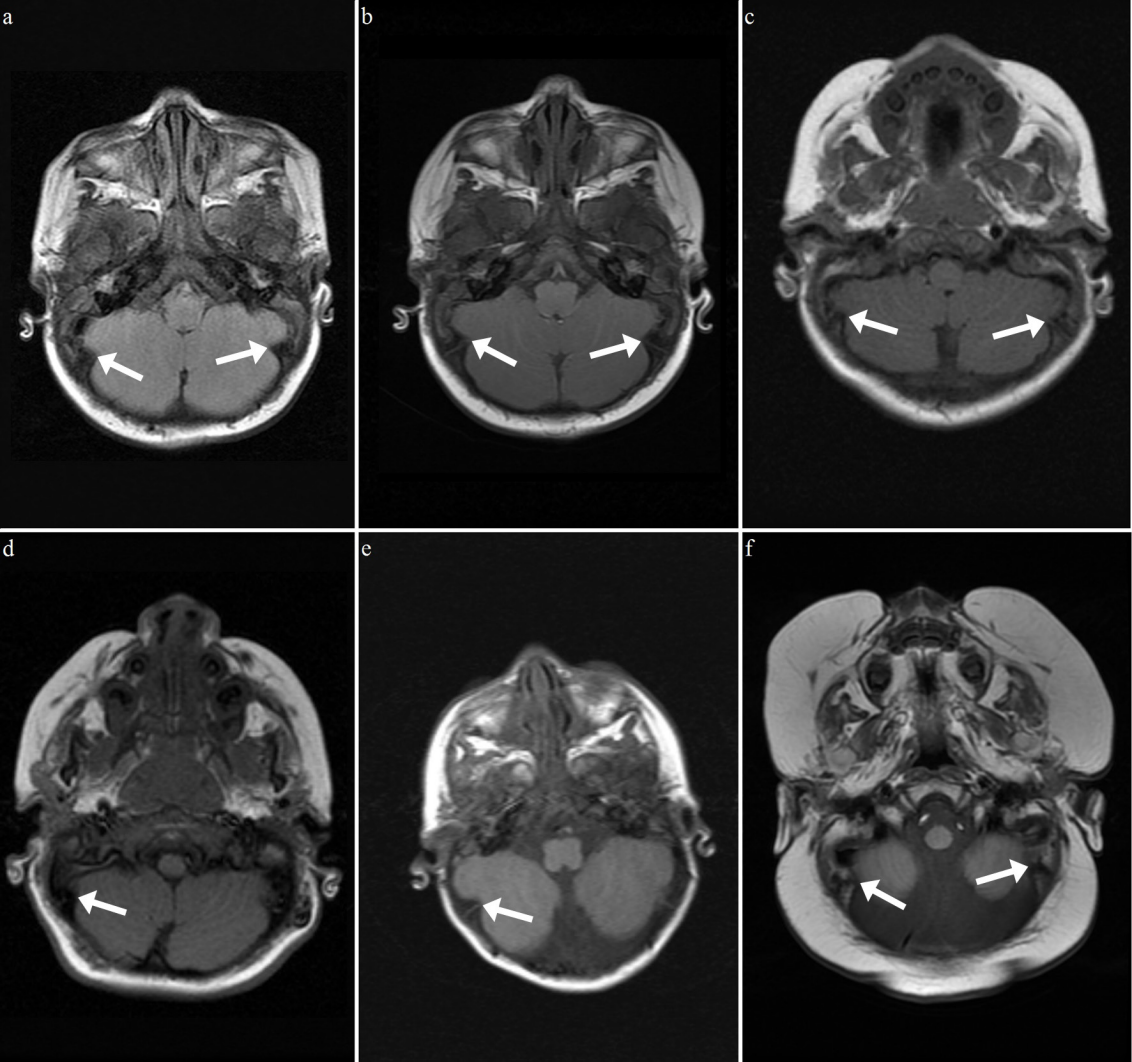
Axial FLAIR (a) and T1 weighted MR images (b–f ) show unilateral or bilateral IHOMs (white arrows) of 6 different patients with MPS type I. Note: MRIs shown in a and b belong to twin siblings. (a: 8 months old, b: 8 months old, c: 13 months old, d: 4 years old, e: 16 months old, f: 23 months old).

**Figure 7:**
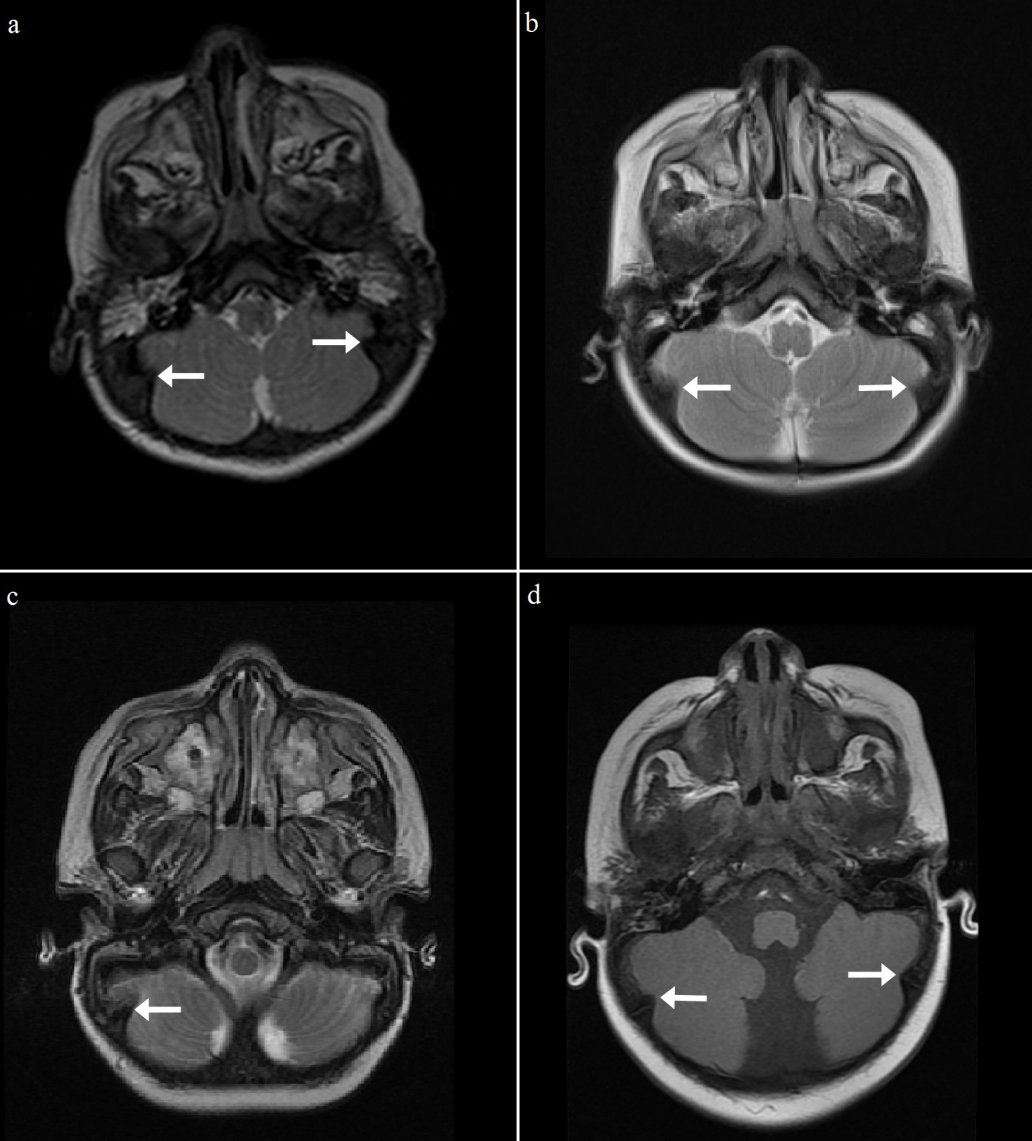
Axial T2 weighted (a–c) and T1 weighted (d) MR images show unilateral or bilateral IHOMs (white arrows) of 4 different patients with MPS type II. (a: 14 months old, b: 15 months old, c: 4 years old, d: 8 months old).

**Figure 8:**
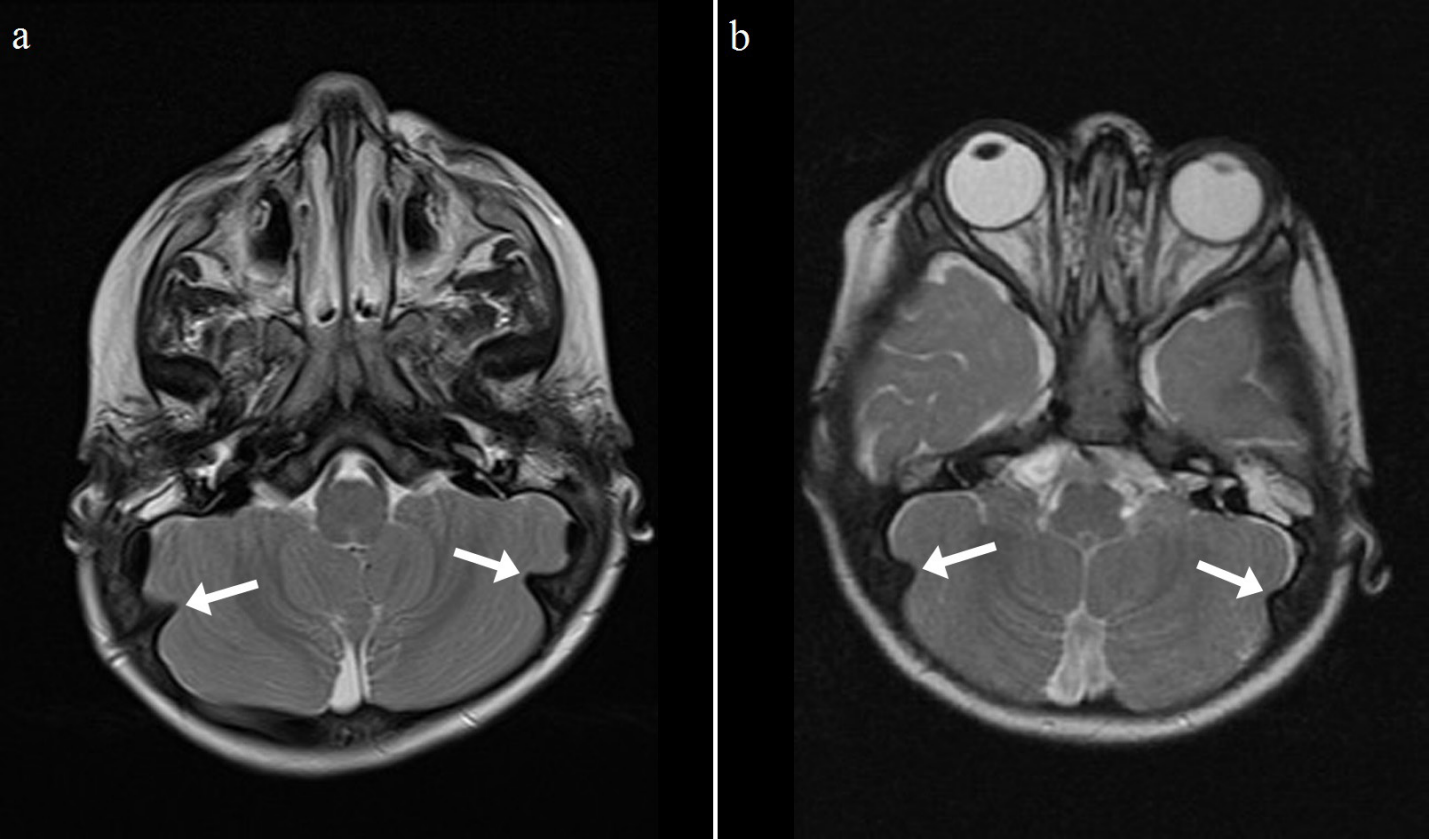
Axial T2 (a-b) weighted MR images show bilateral IHOMs (white arrows) of 2 different patients with MPS type VI. (a: 26 months old, b: 18 months old).

Another calvarial finding is IHOMS. The presence of this finding was evaluated by the consensus of two experienced radiologists. This feature was detected unilaterally (n = 3) or bilaterally (n = 9) in 12 patients, most of whom had MPS type I (n = 6, 54%) (Table 3; Figures 6–8). There are some comments about different posterior fossa findings but this bone peculiarity has never been mentioned in the literature [13,14,17,26]. As discussed above, although remodeling of the cranial bones was encountered in later processes of the disease, most of the IHOMS were detected in younger patients. Five of the 6 patients with MPS type I, 3 of the 4 patients with MPS type II, and both of the patients with MPS type VI were younger than 3 years old. This finding may be related to early cranial suture involvement and GAG deposition. A previous study described that the majority of MPS patients have premature fusion of the cranial sutures [27]. In the present study, none of the patients were evaluated for craniosynostosis. On the other hand, the patients with unilateral IHOMS did not exhibit any posterior cranial deformation suggesting craniosynostosis. According to our observations, this abnormality may be evident with horn-shaped morphology in some MPS patients. There was no significant relationship between calvarial thickening and IHOMS.

**Figure 9:**
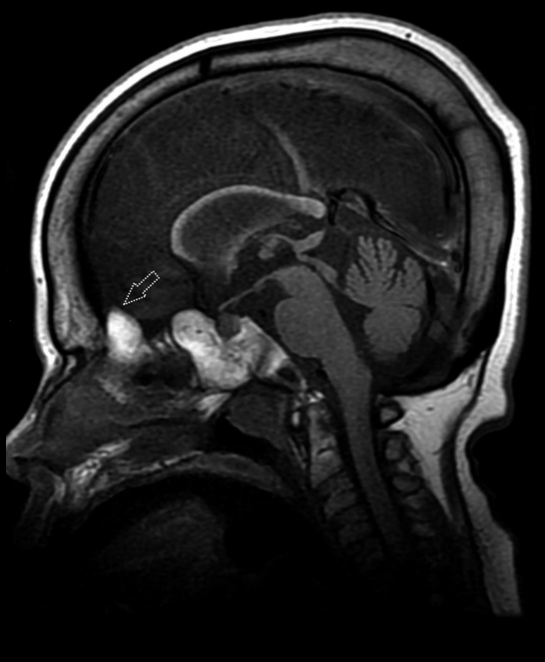
Sagittal T1 weighted MR image shows diffuse calvarial thickening. Note that the lack of pneumatization in frontal and sphenoid sinuses. Hyperintense crista galli has an evident hypertrophic appearance like a white sail (white dotted arrow) (17 years old, MPS type IIIc).

Calvarial thickening with enlargement of diploe spaces was one of the most frequent bone findings in MPS type III patients (16/24 patients, 66%; in all subtypes) [13]. The coarse appearance of the sphenoid body without sinus pneumatization, narrowing of the sella turcica, thick and white (due to presence of fatty bone marrow) sail-shaped crista galli in sagittal T1-weighted images, and lack of pneumatization in bilateral mastoid apex and/or paranasal sinuses were the other evident bone findings that may help to distinguish type III MPS from the other types (Figure 9). 

Together with the clinical and radiological findings, the diagnosis of MPS is possible with quantitative detection of elevated urinary GAG levels. Further studies to detect the specific enzyme deficiencies and genetic analyses are performed to specify the disease type [1,6,28].

Bone marrow transplantations, peripheral blood hematopoietic cell transplantations, umbilical cord blood transplantations, and intravenous/intrathecal enzyme replacement therapies (ERTs) are currently used in the treatment of MPS. ERTs have been used for type I, II, IV, and VI diseases and the production of new enzyme samples has been announced recently for the treatment of type VII disease; studies are still being performed on the use of ERT for type III disease [3,9,12,29]. These treatment options may change the course of the disease and prolong life expectancy, meaning that radiologists may encounter older MPS patients more frequently in the future [9].

There were some limitations of the present study. First, it was a retrospective study and most of these patients had only one MR evaluation and some were lost during follow up. Therefore, there are no specific treatment-related changes in these findings mentioned above, although our center was able to provide treatment for some of the patients who had type I, II, and VI diseases. Second, since some different MRI machines had been used to perform the cranial scans in the present study, the images evaluated were of varying quality, and so the comparability between the MRI images of patients was somewhat limited. Third, there was no three-dimensional constructive interference in steady-state (3D-CISS) studies in cases of ventriculomegaly, arachnoid cysts, and pseudomeningoceles. Fourth, in some patients, because of the difficulties in obtaining quantitative measurements, it was relatively difficult to define hydrocephalus coexistence with cerebral atrophy. Fifth, the skull radiographs were not evaluated for craniosynostosis. Finally, most of MPS subtypes were not known and comparison of the features could not be performed. 

In conclusion, the cranial MRIs of MPS patients frequently exhibit abnormal features. These features may provide diagnostic clues to differentiate the type of the disease in radiological imaging. IHOMS is presented here as a new imaging feature and could also be a helpful finding for diagnosis. Nevertheless, there is a need for further studies of large MPS series to confirm this finding. 

## Acknowledgment

We want to thank all authors of this study.
